# Physical Activity, Physical Fitness, Body Composition, and Nutrition Are Associated with Bone Status in University Students

**DOI:** 10.3390/nu10010061

**Published:** 2018-01-10

**Authors:** Gotzone Hervás, Fátima Ruiz-Litago, Jon Irazusta, Ainhoa Fernández-Atutxa, Ana Belen Fraile-Bermúdez, Idoia Zarrazquin

**Affiliations:** 1 Department of Physiology, Faculty of Medicine and Nursery, University of the Basque Country (UPV/EHU), Bo Sarriena s/n, Leioa, 48940 Bizkaia, Spain; fatima.ruiz@ehu.eus (F.R.-L.); jon.irazusta@ehu.eus (J.I.); 2 Department of Nursing I, Faculty of Medicine and Nursery, University of the Basque Country (UPV/EHU), Bo Sarriena s/n, Leioa, 48940 Bizkaia, Spain; ainhoa.fernandez@ehu.eus (A.F.-A.); anabelen.fraile@ehu.eus (A.B.F.-B.); idoia.zarrazquin@ehu.eus (I.Z.)

**Keywords:** bone health, university students, physical activity, muscle strength, body composition, nutrition

## Abstract

Understanding the modifiable factors that improve and maximize peak bone mass at an early age is necessary to design more effective intervention programs to prevent osteoporosis. To identify these modifiable factors, we analyzed the relationship of physical activity (PA), physical fitness, body composition, and dietary intake with bone stiffness index (SI), measured by quantitative ultrasonometry in young university students (18–21 years). Moderate-to-vigorous PA (MVPA) was the strongest predictor of SI (β = 0.184; *p* = 0.035). SI was most closely related with very vigorous PA in males (β = 0.288; *p* = 0.040) and with the number of steps/day in females (β = 0.319; *p* = 0.002). An association between thigh muscle and SI was consistent in both sexes (β = 0.328; *p* < 0.001). Additionally, extension maximal force was a bone SI predictor factor in females (β = 0.263; *p* = 0.016) independent of thigh muscle perimeter. Calcium intake was the only nutrition parameter that had a positive relationship with SI (*R* = 0.217; *p* = 0.022). However, it was not included as a predictor for SI in our regression models. This study identifies predictors of bone status in each sex and indicates that muscle and bone interrelate with PA and fitness in young adults.

## 1. Introduction

In recent years, osteoporosis has become a serious threat to public health and is thought to be the underlying cause of more than 8.9 million fractures annually worldwide [1]. With the progressive aging of the population, the number of people affected by osteoporosis is rapidly growing. Additionally, hip and vertebrae fractures are significantly associated with high mortality rates [2].

Current treatments for osteoporosis are not entirely effective [3] and simply lead to an increase in health care expenditure. Thus, effective osteoporosis prevention protocols and guidelines aimed at increasing and maintaining bone mass need to be developed. There are many factors, known as environmental influencers, that affect 20–40% of bone accumulation and influence peak bone mass (PBM) reached in early adulthood [4]. PBM is a relevant predictor of bone density in adulthood and old age [5]. Bone mass increases during childhood and puberty but consolidates during young adulthood. In most cases, PBM has already accumulated by age 18–20 years, when maximum bone strength and density is reached. From this point forward, bone mass is reduced, increasing the risk of fracture and osteoporosis. Since bone mass consolidates when PBM is achieved, it is essential to know which modifiable factors improve and maximize the PBM at an early age in order to develop more effective intervention strategies to offset the natural losses caused by aging.

There is evidence that regular physical activity (PA) benefits bone health, contributes to osteoporosis prevention, and may also improve bone mineral density (BMD) [6]. This results in substantial improvements in the mechanical strength of the bone [7]. Intense and repeated activities with rapid changes in direction, accelerations, or jumps produced high forces and unusual or unaccustomed strains, and are key determinants during the growing years for muscle strength and BMD [8].

Self-reported questionnaires to measure PA are unreliable and poorly validated, especially in young populations [9]. Alternatively, accelerometers are widely used to measure the acceleration of the body and to assess the frequency and intensity of PA. Triaxial accelerometers allow PA to be measured objectively by assessing accelerations in three axes (vertical, medio-lateral, and antero-posterior). Consequently, triaxial accelerometers are more sensitive to impact-loading activities, which are beneficial to bone health [10].

In addition to PA, physical fitness—which compromises, at least, cardiorespiratory endurance, muscle endurance, and muscle strength—is an independent predictive factor of certain health outcomes [11]. Both muscular strength and cardiovascular endurance seem to be relevant and linked to the growth and maintenance of bone. Resistance and aerobic exercise interventions result in significant improvements in BMD and bone structural properties during growth [12]. Loading stress causes a modeling response, increasing bone mass and strengthening bones. Moreover, the intensity of exercise can influence the chemical composition of bone and increase BMD.

Body composition is commonly associated with fitness and is correlated with BMD in different skeletal regions [13]. Excess body fat has negative effects on bone mass in adolescents due to elevated levels of adipokines caused by increased bone marrow adiposity [14]. However, increased lean mass is associated with greater bone mass in youths and adults due to mechanical and biochemical coupling between bone and muscle [15]. Skeletal muscle is a secretory organ that produces and releases myokines that have a key protective role in the regulation of bone health [16].

Nutrition is also an important factor in the development and maintenance of bone mass. Calcium and vitamin D intake are essential for the maintenance of bone health [17], however, there are additional dietary contributors such as magnesium, vitamin C, and vitamin K [18,19,20]. Many adolescents consume monotonous, unbalanced, and nutritionally-deficient diets [21] that may reduce PBM. Poor diets developed in childhood frequently persist into adulthood [22], increasing the risk of chronic diseases, such as osteoporosis, later in life. Adopting healthy eating behaviors during the prepubertal period results in increased bone mass and PBM [23].

It is important to identify the factors related to poor bone health in young people to design intervention programs addressing those at high risk of developing osteoporosis in the future. Most studies have evaluated the association of a limited number of variables with bone density. Parameters such as objectively measured PA in the three axes or the strength of specific muscles have not been assessed. We hypothesized that modifiable factors, including body mass and composition, physical activity, muscular and cardiorespiratory fitness, and dietary intake, were associated with bone stiffness at young adulthood. The objective of this study was to investigate the relationship between these modifiable factors and bone stiffness in young university students. We also analyzed which parameters were predictors of bone stiffness in each sex.

## 2. Materials and Methods

### 2.1. Participants

This study was conducted from September 2016 to May 2017. Participants were 156 young adults (61 men and 95 women) aged 18–21 years old (18.74 ± 0.77). Participants were recruited among different university degree programs at the University of the Basque Country (UPV/EHU).

The study was conducted in accordance with the Declaration of Helsinki and was approved by the Research Ethical Committee of the University of the Basque Country (M10/2015/122). The procedures followed were in accordance with the ethical standards of the institutions on human experimentation; all participants participated voluntarily, received verbal and written information about the purpose and procedures of the study, and signed an informed consent document before starting the investigation.

### 2.2. Bone Status

Bone status was measured by non-invasive and easy-to-use quantitative ultrasonometry (QUS). Measurements were performed on the right calcaneus, which has a large percentage of trabecular bone, with a Lunar Achilles Insight ultrasonometer (GE Healthcare, Milwaukee, WI, USA). Correlations between bone parameters measured by ultrasonometry, BMD, and bone mineral content measured by dual-energy X-ray absorptiometry have been observed in children and adolescents [24]. Two different parameters can be assessed by QUS, broadband ultrasound attenuation (BUA, dB/MHz), and speed of sound (SOS, m/s). A third parameter, stiffness index (SI) is calculated by the raw parameters BUA and SOS. SI is the ratio of the traversed distance to the transit time of the ultrasound wave and describes the architecture and connectivity of bone. BUA provides information about bone trabecular complexity and SOS reflects the elasticity of the bone and surrounding soft tissue [25]. The SI variable combines both of these parameters. Consequently, it is the most precise ultrasound variable to measure bone stiffness. Based on the SI, the T-score was estimated. The T-score corresponds to the number of standard deviations from the mean value of the population aged 20–39 years of the same sex.

The manufacturer-provided coefficient of variation for in vivo calcaneus measurement by the QUS Lunar Achilles device is 1.7% [26]. BUA, SOS, and SI measurements assessed by Achilles quantitative ultrasonometry in children and adults provide reproducible results with similar precision [27].

At any rate, SI has been proposed as a suitable QUS parameter to analyze bone health. The better the bone structure, the more sound waves are absorbed, and the higher the SI value. Calibration was carried out before each screening session according to the standard procedure given by the manufacturer.

### 2.3. Anthropometrics

All anthropometric variables were measured according to the protocol recommended by the International Society for the Advancement of Kinanthropometry [28]. Two series of anthropometric measurements were taken on the right side of the body, and the means were used for further calculations.

Height and weight were measured by a stadiometer (Año Sayol, Barcelona, Spain). With a skinfold caliper (Holtain LTD., Crymych, UK), triceps, subscapular, supraspinale, abdominal, thigh, and calf-skinfold thicknesses were estimated. Humerus bicondylar, wrist, femur bicondylar, and ankle girth measurements were taken using a bone anthropometer (Holtain Bicondylar Caliper, Crymych, UK), and arm circumferences while relaxed and flexed as well as thigh and calf circumferences were measured with a tape measure (KaWe, Asperg, Germany). Body mass index (BMI) [29], body fat index [30], and body muscle index [31] were calculated using these values and the accepted procedures and formulas described [29,30,31].

### 2.4. Dietary Consumption

To assess dietary intake, participants were instructed to fill out a food record for five days, indicating everything they ate and drank as accurately as possible from Saturday to Wednesday. To facilitate recording, participants were instructed on how to fill out an example model. Daily energy, macronutrient (protein, carbohydrates and lipids), and micronutrient (vitamins and minerals) consumption was calculated using the DIAL program (Department of Nutrition UCM, Alce Ingeniería S. L., Madrid, Spain). DIAL is a validated computerized nutrient analysis program used to assess nutrient calculation and analyze diets [32].

### 2.5. Strength Measurements

Concentric knee extensor and flexor muscle strength was assessed using an isokinetic dynamometer (HUMAC NORM Isokinetic Extremity System, Stoughton, MA, USA). The isokinetic method assesses muscle strength as the maximal force at muscular contraction. Knee extension and flexion were measured at a 90°/s angular velocity. This is a comfortable speed at which all participants best carried out the test, regardless of whether or not they participated in sports.

The participant’s right thigh was securely strapped to the seat and the joint line of the knee was positioned in line with the axis of rotation of the dynamometer head. To ensure torso stability, a strap was placed across the waist and chest. To maintain ankle anterior/posterior restraint, a pad was fastened between the malleoli and distal soleus. The participant’s knee was placed at 0°, and the limb was weighted using the gravity correction mode.

Before each test, participants partook in a warm-up session consisting of five no-load repetitions at test velocity and 15 s of rest. They were instructed to push the knee as hard and fast as possible during extension and to pull it during flexion in five concentric repetitions of each set. Only the maximal knee extensor and flexor peak torque (Nm) achieved during the five trials was recorded.

### 2.6. Physical Activity

Participants wore an accelerometer (Actigraph wGT3X-BT, Actigraph, Pensacola, FL, USA) on their right hip and took it off during water-based activities, when showering, at bedtime, and during sports with a high risk of damaging the device for seven consecutive days. The ActiLife 6 (ActiLife 6.11.9., Actigraph, Pensacola, FL, USA) software was used to program, score, and clean the accelerometer data files. Data were considered valid when the participant had wore the accelerometer for five days (during the week and at least one weekend day) for at least ten hours per day. The activity counts were stored at 60 s epochs, and periods of 60 min or more without activity recorded were defined as non-wearing time. The total amount of PA was expressed as counts per min (cpm), and all activity data were averaged over the five-day period.

The overall time spent in PA was calculated using the cut-off points of Freedson Adult VM3 (2011): light (0–2689 cpm), moderate (2689–6166 cpm), vigorous (6166–9642 cpm), and very vigorous (9642 cpm and above). Time spent in moderate-to-vigorous physical activity (MVPA) was calculated as the sum of the time in moderate and vigorous activity. Using acceleration data, activity was characterized by three axis movements (approximating sagittal, frontal, and vertical planes) and number of steps.

### 2.7. Aerobic Capacity

The submaximal Astrand-Rhyming test is used to estimate aerobic capacity (VO_2max_). In this ergometer test, participants pedal at 50 revolutions per min and 50–150 watts of power, depending on their heart rate. When the participant’s heart rate is stable and greater than 120 beats per min, the test is finished. This normally takes place between the fourth and fifth min. Heart rate and power were used to estimate absolute and relative (to body weight) VO_2max_ based on the Astrand-Rhyming nomogram, modified for age and sex.

### 2.8. Statistical Analyses

Data on quantitative ultrasonomety, anthropometric parameters, diet, and accelerometer measurements were reported as mean values ± standard deviation (SD). Normal distribution of each parameter was checked with the Kolmogorov–Smirnov test. To explore possible differences between sexes, descriptive characteristics were compared with the *t*-test for continuous variables and the Mann–Whitney U test for continuous non-normally distributed variables. The relationship among measured anthropometry, accelerometry, isokinetic, aerobic capacity, and nutrition parameters with bone was studied by Pearson correlation. This analysis was adjusted for age and sex in the whole group and by age in each sex.

To examine the capacity of PA, dietary consumption, physical condition, and body composition to predict bone SI, a linear regression (backward method) was used. These analyses were also adjusted for age and sex in the whole group and by age in each sex. All analyses were performed using SPSS Version 21.0 (SPSS, Chicago, IL, USA). A value of *p* < 0.05 was considered statistically significant.

## 3. Results

### 3.1. Participants’ General Characteristics

Significant differences were seen between males and females in anthropometric, isokinetic strength, and nutrition variables (Table 1). Females had significantly lower BMI (*p* = 0.007) and body muscle index (*p* < 0.001) and higher body fat index (*p* < 0.001) and thigh skinfold (*p* < 0.001) than males. They also displayed lower peak torque 90°/s values in extension and flexion (*p* < 0.001) than males. Although the males’ calcium intake (1018 mg/day ± 348) reached the recommended value, the females’ average (814 mg/day ± 206) was less than that recommended by the World Health Organization (1000 mg/day). Moreover, neither males (3.88 mg/day ± 2.38) nor females (3.10 mg/day ± 2.23) reached an adequate vitamin D intake (5 mg/day). Calcium consumption (*p* < 0.001) and vitamin D intake (*p* = 0.021) were higher in males.

Non-significant differences between females and males were observed in age, bone measurements (SI, BUA and SOS), thigh perimeter, accelerometer assessments, and aerobic capacity. A slightly higher rate of very vigorous PA was observed in males (*p* = 0.161).

### 3.2. Association between Anthropometry, Physical Activity, Nutrition, and Bone Status

PA, physical fitness, and nutrition measurements were stratified by sex and significant correlations with SI bone parameter were observed (Table 2). Thigh perimeter showed a positive correlation with SI in females (*p* = 0.004) and males (*p* = 0.003). Moderate (*p* = 0.031), very vigorous PA (*p* = 0.028), MVPA (*p* = 0.010), y-axis PA (*p* = 0.010), and steps/day (*p* = 0.002) were also positively correlated with SI in females. SI in males only correlated with very vigorous PA (*p* = 0.008). SI was positively correlated to extension peak torque of 90°/s in females (*p* < 0.001) and flexion (*p* = 0.013) in males. Relative VO_2max_ (mL/kg/min) also had a positive correlation with SI in males (*p* = 0.014). In the overall group, calcium intake showed a positive correlation with SI (*p* = 0.022).

### 3.3. Stiffness Index Prediction

We applied three models of backward linear regression analysis with anthropometric, PA, aerobic capacity, and muscle strength parameters as independent variables and bone SI as the dependent variable (Table 3). Overall, when analyzing participants as a whole group, thigh skinfold (*p* = 0.008) showed a negative association with bone SI. Age (*p* = 0.081) also showed a trend toward negative association with SI. In contrast, thigh perimeter (*p* < 0.001) and MVPA/day (*p* = 0.035) were positive predictors of bone SI. Very vigorous PA (*p* = 0.096) and aerobic capacity (*p* = 0.079) also demonstrated a positive trend with SI.

Analyzing the results by sex, male SI showed a negative trend in association with thigh skinfold (*p* = 0.081) and a positive trend with thigh perimeter (*p* = 0.060). At the same time, very vigorous PA (*p* = 0.040) was positively associated with SI. In females, the number of steps/day (*p* = 0.002) was more influential than thigh perimeter (*p* = 0.008) and extension peak torque 90°/s (*p* = 0.016). These models explain the 28.1% and 33.1% bone SI variance in males and females, respectively.

## 4. Discussion

In this study, we analyzed the interaction between PA, physical fitness, body composition, and nutrition and calcaneus bone density (measured as SI) in female and male university students. There is a general agreement that PA has a positive influence on bone status [33,34]. However, few studies have objectively measured the duration and intensity of PA and the quantity of movement in three spatial axes [35]. Taking the entire sample into account, very vigorous PA was more strongly associated with higher bone SI than activities of lower intensity. These results support the general consensus that high-intensity activity is important for the effective application of mechanical forces and bone development [36].

The association between very vigorous PA and SI was more evident in males (*R* = 0.397) than in females (*R* = 0.267). However, when females were analyzed separately, MVPA and the number of steps per day were associated with calcaneus bone density. In the backward regression models built with parameters that were significantly correlated with SI, very vigorous PA in males and the number of steps per day in females were maintained as significant predictors of SI in the last equation. These sex-related differences may be due to the fact that physical activities performed by men and women are different. Men usually participate in high-intensity sports, while women more often walk [37,38]. Both very vigorous PA and steps per day were included in the last model as independent predictors of SI when the whole population was analyzed. Therefore, intensity and daily steps should be considered as high priority in the design of intervention programs.

When PA was analyzed in three different axes, activity performed in the y-axis showed the highest correlation with SI of the calcaneus. These results support the importance of impact-loading exercises and bone mass accrual [39]. While bone impact prolonged into adulthood has been reported, the duration of the effects is still in question. Moreover, while exercise intensity is key to effective bone development, the role of impact loading in bone development remains unknown. High impact and weight-bearing exercises have an osteogenic effect in young adults [40,41]. However, PA in the vertical axis remains outside the regression equation when all parameters significantly correlated with SI were included in the model. This may be due to the high correlation between the PA in the vertical axis and steps per day or MVPA (*p* < 0.001).

VO_2max_ was also related to calcaneus stiffness in males and in the whole group. This association could be due to the impact of PA on both bone density and fitness. However, in the whole sample, VO_2max_ was maintained in the last equation of the backward regression model as an independent predictor variable for SI, with values close to significance. This independent relationship between aerobic capacity and SI could be due to the fact that PA measured by accelerometer reflects only current activity. However, VO_2max_ could reflect previous PA (not detected by the accelerometer), which can also have an effect on bone density.

The synergy between bone and muscle mass is seen at different levels. The adaptation of muscle and bone is interdependent; alterations in muscle are temporally related with alterations in bone [42]. Additionally, muscle plays a vital role in developing bone strength [43]. The present study strengthens the idea that muscle contributes to bone parameters. Anthropometric perimeter assessments encompass muscle and adipose tissue, while skinfold measurements are used to characterize subcutaneous fat thickness. Our results show a positive association between thigh perimeter (*p* < 0.001) and SI, and a negative link between thigh skinfold (*p* = 0.008) and SI. These associations were maintained in all models. Thus, higher muscle perimeters are associated with higher calcaneus stiffness. A recent study reported that midthigh muscle volume is moderately-to-strongly related to trabecular bone architecture [44]. In our study and others, the association between muscle mass and bone parameters is consistent in both sexes [45].

Two explanations have been given for this association between muscle mass and bone parameters. There is known muscle-bone cross-talk and muscle-derived myokines exert anabolic effects on bone [3,16]. Thus, in one explanation, an increased muscle mass would lead to a higher secretion of myokines, which can have greater effects on bone density [46]. In the second explanation, mechanical stress during muscular contraction alters muscle strength, which is linked with bone strength [47]. In our study, knee extension maximal muscle force showed an association with SI in females and in the whole sample. In males, on the other hand, we found an association between SI and flexion maximal force. In females, the extension maximal force was included in the regression model built with factors significantly correlated with SI (*p* = 0.016), independent from thigh fold and perimeter. Therefore, the results of this study suggest that both muscle mass and strength are independent predictor variables of SI.

The young university students participating in this study had unbalanced diets. This is consistent with other studies showing that young persons’ dietary habits are not aligned with national recommendations [21,48]. Vitamin D and calcium intake were higher in males than in females. This may be due to the fact that males eat more than females overall and thus have an increased vitamin and mineral intake. Our study only showed a significantly positive relationship between calcium intake and SI when taking the whole group into account. The influence of calcium on young adults’ bone parameters can be compensated for by the effects of physical activity. Nevertheless, it is worth highlighting that unbalanced diets limit mineral and vitamin intake, which hinders our ability to see the relationship between vitamin D and calcium intake with bone stiffness.

There are some additional limitations on this research that should be considered. Due to the cross-sectional nature of this study, causality cannot be inferred from our results. In addition, bone stiffness is different at each location. This means that the measurement of bone stiffness provides information about the location where it was assessed. Moreover, this technique does not analyze bone mass, density, and geometry separately, giving only an estimation of bone mineral status. Nonetheless, the simplicity, versatility, low cost, and lack of ionizing radiation of QUS have led to the diffusion of this method worldwide. Furthermore, records of dietary intake were self-reported. Thus, it must be taken into consideration that participants tend to underestimate the consumption of food with a negative health image and over-report foods with a positive health image [49]. Moreover, the conclusions drawn from this sample of university students cannot be applied directly to other populations. However, this limitation does not preclude reliable results.

The strengths of this study include that, to date, we are not aware of any studies in which the association between bone stiffness and muscle strength in young adults has been analyzed in based on physical activity and physical fitness as measured by accelerometry, aerobic capacity, and isokinetic dynamometry. The use of accelerometers gives more weight and credibility to the study than earlier studies in which PA was measured using subjective questionnaires. Furthermore, we analyzed a number of variables that could affect bone stiffness, such as cardiorespiratory fitness, muscle strength, anthropometry, and dietary intake.

## 5. Conclusions

PA and nutrition are important bone health contributors that have been established as important modifiable factors influencing bone mineral accrual and maintenance. MVPA and very vigorous PA are positively related to bone stiffness in the whole sample. However, the associations are different depending on sex. Thus, SI in males showed more association with very vigorous PA and SI in females presented the largest association with steps/day. Furthermore, an association between body composition and bone stiffness was evident. Our findings suggest a consistent association between thigh muscle, MVPA, and bone stiffness. By identifying which contributors are most important to enhance bone SI, more efficient prevention programs could be conducted from an early age.

## Figures and Tables

**Table 1:** Descriptive characteristics of participants and *p*-values for comparison by sex.

Characteristics	Females (*n* = 95)	Males (*n* = 61)	Overall (*n* = 156)	*p*-Value
Age (years)	18.7 ± 0.8	18.8 ± 0.8	18.7 ± 0.8	0.667 ^b^
Bone measurements				
SI	115 ± 17	119 ± 24	117 ± 20	0.290 ^a^
BUA (dB/MHz)	120 ± 14	124 ± 16	122 ± 15	0.065 ^a^
SOS (m/s)	1626 ± 45	1634 ± 71	1629 ± 57	0.107 ^b^
T-score	1.39 ± 1.35	1.53 ± 1.80	1.44 ± 1.54	0.563 ^a^
Anthropometry				
Thigh skinfold (mm)	31.0 ± 6.7	23.6 ± 7.3	28.1 ± 7.8	<0.001 ^b^
Thigh perimeter (cm)	50.4 ± 4.4	50.8 ± 3.8	50.5 ± 4.2	0.493 ^a^
BMI (kg/m^2^)	21.7 ± 2.5	22.7 ± 1.9	22.1 ± 2.4	0.007 ^a^
Body fat index (%)	21.1 ± 4.1	12.8 ± 3.0	17.8 ± 5.5	<0.001 ^a^
Body muscle index (%)	41.6 ± 4.6	49.8 ± 3.1	44.8 ± 5.7	<0.001 ^a^
Accelerometry				
Light/day (min)	791 ± 129	775 ± 76	785 ± 111	0.935 ^b^
Moderate/day (min)	57.2 ± 19.1	57.7 ± 17.3	57.4 ± 18.9	0.892 ^a^
Vigorous/day (min)	8.90 ± 8.94	10.2 ± 9.23	9.38 ± 9.04	0.432 ^b^
Very vigorous/day (min)	1.59 ± 4.02	2.22 ± 2.93	1.83 ± 3.64	0.161 ^b^
MVPA/day (min)	67.7 ± 25.2	70.0 ± 24.0	68.6 ± 24.7	0.687 ^b^
Sedentary time /day (min)	403 ± 128	408 ± 85	405 ±113	0.463 ^b^
Y-axis (cpm)	66.6 ± 25.7	70.7 ± 25.9	68.1 ± 25.8	0.361 ^b^
X-axis (cpm)	56.3 ± 20.1	54.7 ± 16.2	55.6 ± 18.6	0.650 ^a^
Z-axis (cpm)	60.5 ± 19.6	63.4 ± 21.1	61.6 ± 20.1	0.471 ^b^
Steps/day	9339 ± 2752	9196 ± 2734	9284 ± 2735	0.780 ^a^
Isokinetic assessment				
PT_EX90_ (Nm)	114 ± 34	174 ± 52	137 ± 51	<0.001 ^b^
PT_FLX90_ (Nm)	61.4 ± 15.4	103 ± 23	77.1 ± 27.5	<0.001 ^b^
Aerobic capacity				
VO_2max rel_ (mL/kg/min)	54.8 ± 10.3	58.1 ± 12.7	56.1 ± 11.3	0.158 ^b^
Nutrition				
Energy (kcal)	2259 ± 541	2761 ± 631	2453 ± 625	<0.001 ^a^
Protein (%)	22.1 ± 3.3	22.5 ± 3.4	22.2 ± 3.3	0.509 ^a^
Carbohydrate (%)	54.2 ± 5.9	53.3 ± 6.3	53.8 ± 6.0	0.411 ^a^
Lipid (%)	23.8 ± 4.3	24.3 ± 4.3	23.9 ± 4.3	0.524 ^a^
Calcium (mg)	814 ± 206	1018 ± 348	893 ± 287	<0.001 ^a^
Vitamin D (mg)	3.10 ± 2.23	3.88 ± 2.38	3.40 ± 2.32	0.021 ^b^

Data are shown as mean ± SD. SI: stiffness index; BMI: body mass index; BUA: broadband ultrasound attenuation; SOS: speed of sound; BMI: body mass index; PT_EX_: peak torque extension; PT_FLX_: peak torque flexion; MVPA: moderate to vigorous physical activity; cpm: counts per min; VO_2max rel_: relative aerobic capacity; ^a^ Student’s *t*-test; ^b^ Mann–Whitney U test.

**Table 2:** Correlations between SI and anthropometric, physical activity, physical fitness, and nutrition variables.

Variables	Females ^a^		Males ^a^		Overall ^b^	
	*R*	*p*-Value	*R*	*p*-Value	*R*	*p*-Value
Thigh skinfold (mm)	−0.116	0.348	−0.248	0.105	−0.174	0.067
Thigh perimeter (cm)	0.342	0.004	0.433	0.003	0.356	<0.001
BMI (kg/m^2^)	0.186	0.128	0.136	0.379	0.153	0.108
Body fat index (%)	−0.031	0.805	−0.264	0.084	−0.121	0.202
Body muscle index (%)	0.109	0.377	0.214	0.162	0.132	0.165
Light/day (min)	0.091	0.443	−0.085	0.574	0.026	0.777
Moderate/day (min)	0.262	0.031	0.190	0.216	0.222	0.019
Vigorous/day (min)	0.174	0.156	0.153	0.320	0.163	0.087
Very vigorous/day (min)	0.267	0.028	0.397	0.008	0.303	0.001
MVPA/day (min)	0.310	0.010	0.245	0.109	0.274	0.003
Sedentary time/day (min)	−0.036	0.773	−0.160	0.300	−0.079	0.409
Y-axis (cpm)	0.311	0.010	0.179	0.245	0.238	0.012
X-axis (cpm)	0.188	0.124	0.175	0.256	0.172	0.070
Z-axis (cpm)	0.201	0.100	0.188	0.222	0.196	0.039
Steps/day	0.366	0.002	0.170	0.271	0.265	0.005
PT_EX90_ (Nm)	0.419	<0.001	0.109	0.481	0.251	0.007
PT_FLX90_ (Nm)	0.098	0.424	0.372	0.013	0.141	0.138
VO_2max rel_ (mL/kg/min)	0.088	0.397	0.316	0.014	0.201	0.010
Calcium (mg)	0.151	0.218	0.258	0.091	0.217	0.022
Vitamin D (mg)	−0.006	0.960	0.101	0.514	0.050	0.599

SI: stiffness index; BMI: body mass index; PT_EX_: peak torque extension; PT_FLX_: peak torque flexion; MVPA: moderate to vigorous physical activity; cpm: counts per min; VO_2max rel_: relative aerobic capacity. ^a^ Corrected for age; ^b^ Corrected for age and sex.

**Table 3:** Backward regression coefficients, R^2^ and *p*-values for regression analyses predicting SI.

	Females ^b^				Males ^b^				Overall ^a^			
	B	Beta	*p*-Value	*R*^2^	B	Beta	*p*-Value	*R*^2^	B	Beta	*p*-Value	*R*^2^
Age (years)	−5.734	−0.264	0.052	0.331	-	-	-	0.281	−3.849	−0.143	0.081	0.274
Thigh skinfold (mm)	-	-	-		−0.792	−0.231	0.081		−0.602	−0.229	0.008	
Thigh perimeter (cm)	0.793	0.209	0.008		2.133	0.270	0.060		1.672	0.328	<0.001	
Very Vigorous/day (cpm)	-	-	-		2.501	0.288	0.040		0.798	0.145	0.096	
MVPA/day (cpm)	-	-	-		-	-	-		0.154	0.184	0.035	
Steps/day	0.002	0.319	0.002		-	-	-		-	-	-	
PT_EX90_ (Nm)	0.185	0.263	0.016		-	-	-		-	-	-	
PT_FLX90_ (Nm)	-	-	-		-	-	-		-	-	-	
VO_2 max rel_ (mL/kg/min)	-	-	-		0.489	0.228	0.096		0.279	0.147	0.079	

B: unstandardized regression coefficient; Beta: standardized regression coefficient; SI: stiffness index; PT_EX_: peak torque extension; PT_FLX_: peak torque flexion; MVPA: moderate to vigorous physical activity; cpm: counts per min. ^a^ Corrected for age ^b^ Corrected for age and sex.
